# Understanding the Role of Misinformation in COVID-19 Vaccine Hesitancy in a Rural State

**DOI:** 10.3390/vaccines10050818

**Published:** 2022-05-21

**Authors:** Ann Marie R. Hess, Colin T. Waters, Elizabeth A. Jacobs, Kerri L. Barton, Kathleen M. Fairfield

**Affiliations:** 1 Maine Medical Center, Maine Health, Portland, ME 04101, USA; eajacobs1@mmc.org (E.A.J.); fairfk@mmc.org (K.M.F.); 2 College of Health Sciences, Rush University, Chicago, IL 60612, USA; 3 School of Medicine, Tufts University, Medford, MA 02111, USA; colin.waters@tufts.edu; 4 Maine Medical Center Research Institute, Scarborough, ME 04074, USA; 5 Public Health Division, Portland, ME 04101, USA; kbarton@portlandmaine.gov

**Keywords:** vaccine hesitancy, vaccine misinformation, COVID-19 vaccine hesitancy

## Abstract

Objective: to identify factors associated with COVID19 vaccine hesitancy, including sources of information among residents of Maine. Methods: 148 study participants, recruited through community partners and primary care offices in Maine, completed an anonymous 15 item online survey. Recruitment and data collection occurred from May to September, 2021. Hesitancy was determined through a single question, “Will you get one of the COVID vaccines when it is offered to you?” Results: vaccine hesitant respondents were younger than not hesitant respondents (*p* = 0.01). Hesitant individuals were significantly more likely to report concerns regarding the speed of COVID-19 vaccine production, vaccine efficacy, and potential vaccine side effects (*p* < 0.05 for each). Hesitant individuals were also significantly more likely to have discussed vaccination with their primary physician (*p* = 0.04). Conclusions: overall, hesitant individuals are more likely to be younger and had less trust in information from government sources, but they sought input from primary care. They were also more concerned about efficacy, side effects, and the rapid development of COVID-19 vaccines. Primary care physicians are in key positions to address these concerns due to contact with individuals who need accurate information.

## 1. Introduction

The coronavirus pandemic remains a threat to public health worldwide. Vaccination remains the most effective measure for reducing hospitalizations and deaths and for mitigating the global impact of COVID-19. Vaccinations have been available in the United States since December of 2020, and FDA approvals continue to increase public access to the vaccine. Despite efforts to increase vaccine uptake and distribution, the percentage of people fully vaccinated in the United States was 59.7% with a seven day case rate of 203/100 k, as of 3 December 2021 [1]. Targeted efforts to increase vaccine uptake are, therefore, critical. Vaccination against COVID-19 and achieving normality has proven to be challenging politically and logistically [2,3]. In past studies, public concerns regarding the safety and side effects of other vaccines, as well as perceptions of vaccine safety and efficacy, were the strongest predictors of overall uptake [4,5]. Large scale cross-sectional studies have reported factors associated with the intention to get the COVID-19 vaccine, including efficacy, protection duration, perceived COVID-19 risk, short and long-term side effects, speed of the vaccine approval process, origin of the vaccine, and sources of information and endorsement [6,7,8]. COVID-19 vaccine hesitancy is also associated with exposure to misinformation and political affiliation [9].

Rural Americans may have different beliefs about contracting COVID-19, as well as unique risks for adverse outcomes, due to poverty and lack of access to health care [10,11]. Prior work in rural communities found higher COVID-19 vaccine hesitancy, lower vaccination rates, and lower trust in the government as a source of information [6,12,13,14,15].

Several studies have identified other demographic variables associated with a lower likelihood of getting the vaccine, including having no college degree and being female and white [6,16,17]. Acceptance of the COVID-19 vaccine varies substantially across different sectors of the American population and is changing over time. Surveys among Americans showed the highest intended acceptance was in early April of 2020, dropping significantly by October of 2020, and rising again in February of 2021 [1]. In September of 2021, the Kaiser Family Foundation reported the Delta variant and full FDA approval of the vaccines to be key motivators for individuals to get vaccinated. Vaccine mandates, however, showed minor influence on vaccine intention [18].

Additional factors associated with increased confidence in getting vaccinated against COVID-19 include receiving encouragement from a personal physician and opinions of families and friends supporting vaccination [7,19]. Verger and Dube [20] found an association between confidence in the vaccine with trust in professionals and science. The influence of politics has played an unprecedented role in COVID-19 vaccine hesitancy, with marked differences between Democrats and Republicans [21], possibly due to misinformation such as conspiracy theories and lack of trust in government and healthcare professionals [8].

A “3C” model of influenza vaccine hesitancy, described by Larson et al. [22], was recently expanded to a “5C” construct by Razai et al. [23] The model defines five factors that influence vaccine hesitancy: (1) confidence, (2) complacency, (3) convenience, (4) communications or sources of information, and (5) context or sociodemographic status [23]. *Confidence* focuses on attitudes, beliefs, and concerns about vaccine effectiveness, efficacy, and safety [22,24]. Kreps et al. [7] further explained confidence as whether a vaccine received full US Food and Drug Administration (FDA) approval and whether the source of vaccine endorsement was trusted. Dube et al. [25] suggested that confidence in vaccines depends on trust in science and professionals in a socio-political context. *Complacency* describes individuals who do not perceive a value or need for getting a vaccine. *Convenience* is associated with ease of access to vaccines [22,24]. *Communications* focuses on sources of rapidly changing guidance and the spread of false information about COVID-19. *Context* reflects sociodemographic constructs such as age, sex, and education.

Our aim was to use the “5C” model to describe factors contributing to vaccine hesitancy and uptake intention from a sample of residents in Maine, one of the most rural states in the country. This was a descriptive study, and no hypothesis was developed or tested.

## 2. Methods

We developed a 15-item survey in Qualtrics, designed according to the “5C” construct, to explain vaccine hesitancy [23]. To assess confidence among Maine residents, we measured participants’ trusted sources of information, trust in science and professionals, concerns about side effects, how well the vaccine works, and trust in the vaccine approval process. We measured complacency via examining beliefs about protecting one’s self and others, personal experience with COVID-19 infection, and political affiliation. To assess convenience, we measured levels of concern about where vaccines were being administered among survey respondents. Lastly, we queried respondents about trusted sources of information about the virus and vaccines. Demographic data (context) were collected for age, sex, race, ethnicity, education, and county of residence. The survey combined questions from different entities and provided discrete response categories. Four questions assessed vaccine concerns using a four point Likert scale. Comment sections were added to allow elaboration regarding trusted sources of information, concerns, vaccine preference, motivations, and plans for getting the vaccine. Survey data were de-identified to ensure anonymity and confidentiality during data collection. The survey instrument was not validated prior to implementation.

Three community partners were eager to support participant recruitment and provide input on all aspects of the research. The efforts of the Northern New England Clinical and Translational Research network Rural Core established our partners, including Healthy Community Coalition (HCC) of Greater Franklin County, Pen Bay Community Health and Wellness (CHW), and Healthy Oxford Hills (HOH) located in Norway, Maine. Providers from three primary care practices volunteered to participate in the recruitment of survey participants with interest in results reflecting their populations. Study flyers were used to support in-person, email, and social media recruitment and communication. Instructions included a web link for access to a survey administered via REDCap. A contact number was provided for completing the survey via telephone with a research coordinator; however, all respondents used the web address. The study design did not include interviews, and there were no incentives or gifts for participation.

Recruitment and data collection, through the methods described for this descriptive study design, used a convenience sample of people in Maine and occurred over four months from 27 May 2021 to 21 September 2021. We categorized people as “hesitant” or “not hesitant” by their response to the question “Will you get one of the COVID-19 vaccines when it is offered to you?” Hesitant respondents were categorized by a response of “I do not plan to get the vaccine”, “I am not sure what I will do”, or “I will wait and see what happens.” Not hesitant respondents were those with responses including “I will definitely get a vaccine”, “I will probably get a vaccine”, “I got the vaccine”, or “I already got the vaccine”. Rurality was defined using the 2013 USDA Urban Influence Codes to distinguish metro from non-metro counties in the State of Maine [26].

We used Fisher’s exact test to analyze demographic data, and the Wilcoxon rank sum test was used to compare differences between groups in concerns over vaccine effectiveness, side effects, and speed of production, as well as concerns about traveling to be vaccinated. Metro counties were defined as Androscoggin, Cumberland, Penobscot, Sagadahoc, and York using the 2013 USDA Urban Influence Codes [26]. Analysis was conducted in R version 3.2.4 for Apple x86-64. Survey entries were manually reviewed and cleaned for a total of 148 respondents in the analytic file, removing two redundant entries with identical responses and similar time-stamps.

## 3. Results

The majority of respondents reported being female, white, aged 35 years or older, from metropolitan areas, educated at the college level or higher, affiliation with Democrats, and were vaccinated or planning to be vaccinated (Table 1). Fourteen of 148 (9.5%) were hesitant. The hesitant and not hesitant groups were significantly different in age (*p*-value = 0.01) (Table 1), with a greater proportion of the hesitant group between 35 and 54 years of age (57% vs. 25%) and a greater proportion of the not hesitant group were 65 years of age and older (31% vs. 0%). A significant difference in political affiliation was also found when comparing hesitant vs. not hesitant respondents (*p*-value < 0.001). There was no significant difference in reported vaccine hesitancy between male and female respondents (*p*-value = 0.07), metro and non-metro county of residence (*p*-value = 0.09), education level (*p*-value = 0.12), or by self-reported race and ethnicity (*p*-value = 1).

**Figure 1:**
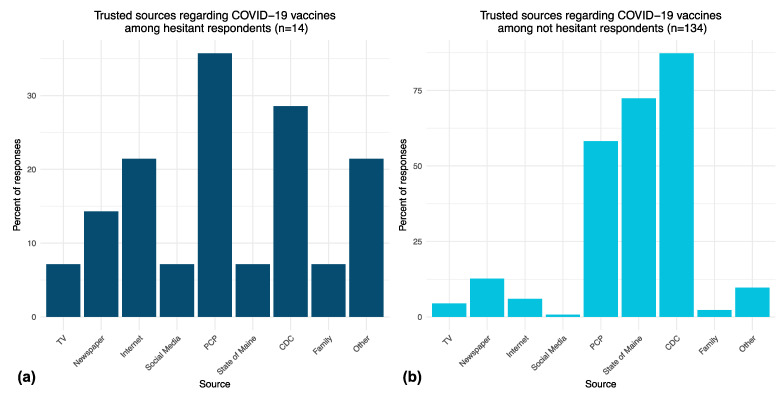
(**a**) Percentage of responses related to trusted sources of information about COVID-19 vaccines among hesitant respondents. (**b**) Trusted sources of information among not hesitant respondents.

The top trusted sources of information on COVID-19 vaccines differed somewhat between hesitant and not hesitant respondents (Figure 1). When asked to pick three trusted sources on COVID-19 vaccines, hesitant respondents most frequently reported trust in their PCP (36% of respondents), the CDC (29% of respondents), and “other” sources (21% of respondents) (Figure 1a). Not hesitant respondents most frequently reported trust in the CDC (87% of respondents), the State of Maine website (72% of respondents), and their PCP (58% of respondents) for information on COVID-19 vaccines (Figure 1b).

**Figure 2:**
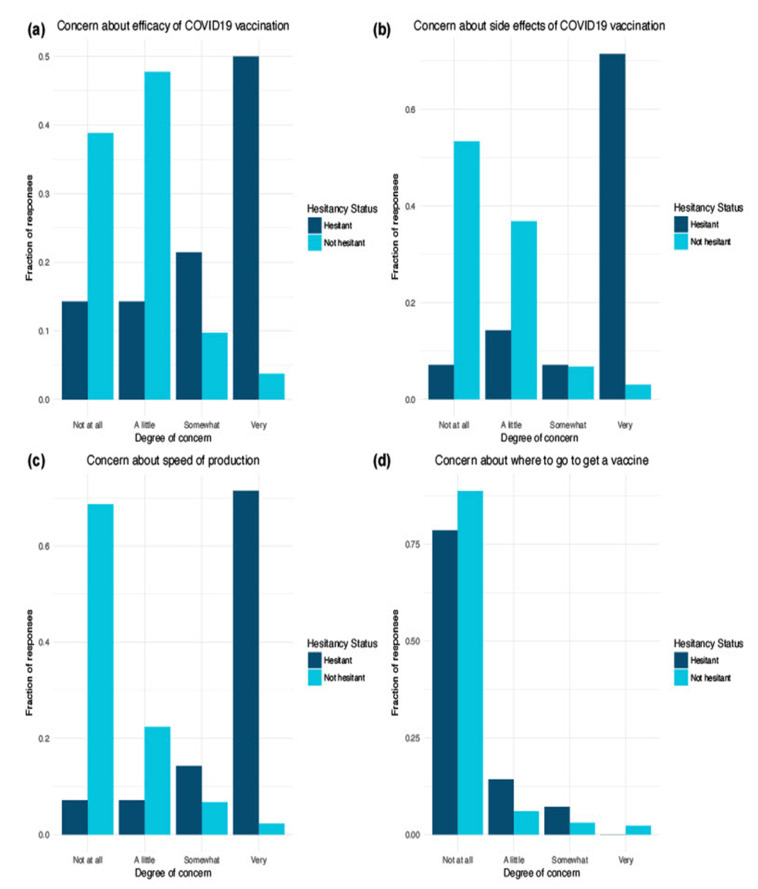
(**a**) Degree of concern between hesitant and not hesitant respondents about efficacy of COVID19 vaccination. (**b**) Degree of concern about side effects of COVID19 vaccination. (**c**) Degree of concern about COVID19 vaccine speed of production. (**d**) Degree of concern about where to get a COVID19 vaccine.

We identified several differences between hesitant and not hesitant respondents related to concerns about COVID-19 vaccines (Figure 2). Hesitant respondents were more likely to be concerned about the efficacy of COVID-19 vaccines than the not hesitant group (*p*-value < 0.05) (Figure 2a). Hesitant respondents were more likely to be concerned about potential COVID-19 vaccine side effects (*p*-value < 0.05) (Figure 2b).

The comments by women of child bearing age revealed misinformation about side effects (Table 2). For example, “I have heard a lot of women saying their cycles have been irregular after the vaccine, although the CDC says it doesn’t impact fertility, I am skeptical because of this.” Multiple comments specified worries about the vaccine causing medical conditions to worsen. A vaccinated respondent said, “I am worried it has increased my already present health conditions into something more quickly progressive (still, better than a ventilator and death).” Hesitant respondents were more likely to be concerned about the speed of production of the COVID-19 vaccine (*p*-value < 0.05). A respondent shared concerns about a rushed process, possibly influenced by misinformation, stating, “I’m scared of COVID but I am also scared of the vaccine. I have researched each one extensively and I still believe we have somewhat rushed the process.” Another respondent believed “it should take 20 years plus to test” a vaccine. Respondents were highly vocal regarding the construct of confidence, with over 200 comments providing a deeper understanding of concerns driving hesitancy to inform strategies to address misinformation.

**Figure 3:**
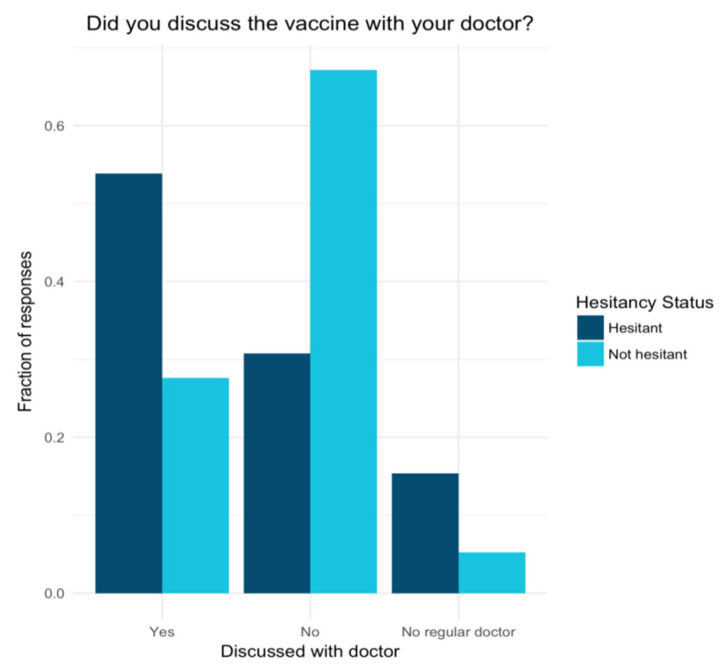
Hesitant and not hesitant group comparisons of whether they discussed the vaccine with a doctor.

No significant difference was found between the two groups regarding knowledge of where to obtain a vaccine (*p*-value = 0.3), with 79% of hesitant and 89% of not hesitant respondents “not at all” concerned about where to go to get a vaccine. Finally, hesitant respondents were significantly more likely to have discussed the vaccine with their doctor than not hesitant respondents (*p*-value = 0.04) (Figure 3).

## 4. Discussion

We found several differences between hesitant and not hesitant residents of a rural state, during a period of evolving disease prevalence and vaccine access, including trusted sources of information. Hesitant respondents were significantly more likely to endorse concerns about the efficacy of COVID-19 vaccination, potential side effects of COVID-19 vaccination, and the speed with which COVID-19 vaccines were produced.

Overall, we find increased hesitancy among younger individuals, consistent with previous studies, showing that older adults tend to prioritize the benefit of reducing serious illness over the cost of side effects [21]. This may reflect an initial prioritization of adults 65 years and older as eligible for vaccination. Furthermore, age is frequently cited in the scientific literature and lay press as contributing to the morbidity and mortality of COVID-19 infection, which may have an influence on decision making [27]. We also found a significant difference in political affiliation between hesitant and not hesitant respondents, with hesitant respondents more frequently reporting being independents or unaffiliated versus not hesitant respondents reporting affiliation with the Democratic Party.

Notably, we did not find a significant difference in hesitancy when comparing metro and non-metro respondents, suggesting that strategies employed to increase uptake in urban areas could be employed in a rural setting as well. Additionally, we found that family and friends are trusted sources of information. These networks could be leveraged more effectively to address concerns related to COVID-19 vaccination efficacy, side effects, and speed of production. However, vaccine misinformation may also spread in these networks. As identified in prior work, the effects of misinformation and political polarization should inform all strategies to increase vaccination rates [9].

Assumptions about the finding that hesitant respondents were more likely to discuss vaccination with their primary care provider include: (a) not hesitant respondents had already made up their minds regarding the vaccine and, therefore, did not solicit more information from their physicians, or (b) respondents in the hesitant group may be eager to pursue information on vaccinations, but overall, they are more skeptical of conventional or government sources, such as physicians, the CDC, and the State of Maine. Moreover, primary care providers were the most frequently reported trusted source of information on COVID-19 vaccines in the hesitant group and greater than media sources, the CDC, and the State of Maine. These data suggest health care providers may be in a key position to address misinformation about vaccines and address patient concerns about getting the vaccine. A national survey of 9000 adults, ages 18–64, reported that two-thirds had a personal doctor, and three in four of these adults trusted health care providers for information. However, only one in five had obtained such information from their personal doctor. These findings suggest a greater need for advancing the role and training of health care professionals to address lingering concerns of COVID-19 vaccine hesitant individuals [28].

Study limitations include convenience sampling, a small sample size for the not hesitant group, lack of racial and ethnic diversity, high levels of participant education, and increases in availability of the vaccine during the study period. Data were collected before the Delta and Omicron virus surges, which may have positively impacted vaccine intention, as recently reported by Kaiser Family Foundation.^18^ Low and unequal representation of Republican Party affiliation (9%) could be a limitation of hesitancy findings associated with politicization. Comparison of metro and non-metro groups was limited by similar demographics across groups, potentially impacting significance of the analysis.

## 5. Public Health Implications

Findings from this study, regarding vaccine hesitancy and sources of information, are important for developing public health strategies to increase vaccination rates for successful mitigation of the COVID-19 pandemic. Vaccine hesitant individuals comprise one end of a continuum, ranging from total acceptors to complete refusers. Understanding the concerns of individuals, as assessed in this study, provides opportunities to address misinformation, target messaging, and influence the ‘movable middle’ toward getting the vaccine [25,29]. Our study reveals that vaccine hesitant individuals have specific concerns about COVID-19 vaccinations, such as worries about existing conditions and side effects. Trained and trusted health professionals, as well as family and friends who need accurate information as trusted sources, could effectively address such concerns and misinformation. Women of child bearing age were particularly vocal about a need for information from trusted sources. The findings from this study emphasize the role of health care providers as sought out and trusted resources for guidance. Providers are uniquely positioned to influence the hesitant middle of the continuum, particularly by addressing worries about the effect of vaccines on existing medical conditions. The findings from this study in Maine communities will inform improvements in outreach and messaging strategies. Leveraging the role of health professionals to encourage vaccine acceptance should continue to focus on training to address misinformation and on updating materials for health professionals to share during visits. 

## Figures and Tables

**Table 1:** Characteristics of Survey Participants and Comparison Hesitant and Not Hesitant Group Characteristics (*N* = 148).

	Total N (%)	Hesitant N (%)	Not Hesitant (N%)	Fisher Exact
**Total N**	**148**	**14**	**134**	
**Age Range**				
18 to 34 years	26 (18%)	3(21%)	23(17%)	0.01
35 to 54 years	42 (28%)	8(57%)	34(25%)	
55 to 64 years	38 (26%)	3(21%)	35(26%)	
65 and older years	41 (28%)		41(31%)	
Unknown			1(1%)	
**Sex**				
Female	124 (84%)	9(64%)	115(86%)	0.07
Male	19 (13%)	4(29%)	15(11%)	
Other or prefer not to answer	5 (3%)	1(7%)	4(3%)	
**Race/Ethnicity**				1.0
White	136 (92%)	13(93%)	123(92%)	
Hispanic or Latinx	3 (2%)		3(2%)	
**Rurality (17 Counties)**				
Metro	76 (51%)	4(29%)	72(54%)	0.09
Non-Metro	65 (44%)	9(64%)	56(42%)	
**Education**				0.12
High School Graduate or Less	9 (6%)	3(21%)	6(4%)	
Some College	19 (13%)	2(14%)	17(13%)	
Four Year Degree	42 (28%)	3(21%)	39(29%)	
Post Graduate	78 (53%)	6(43%)	72(54%)	
**Political Affiliation**				
Democrat	88 (59%)		88(66%)	<0.001
Republican	13 (9%)	2(14%)	11(8%)	
Independent	25 (17%)	6(43%)	19(14%)	
No Affiliation	22 (15%)	6(43%)	16(12%)	
**Vaccination Status**				
Vaccinated or Plan to Be	134 (84%)			
Vaccine hesitant	14 (9%)			

**Table 2:** Comments by Construct.

Construct (5Cs)	Survey Category	Comments
Communication Source of Information	Info about Virus or Vaccine	Researching myself using a variety of sources. My own ability to decipher the numbers we are given. Looking at data from the CDC and current research myself. I have heard a lot of women saying their cycles have been irregular after the vaccine, so though the CDC says it doesn’t impact fertility, I am skeptical because of this. I was actively trying to get pregnant when I received the vaccine and was somewhat concerned an inflammatory cascade might cause a very early spontaneous abortion or affect fertilization. I feel this vaccine is pushed by the media and government. People are being “peer pressured”. Herd Immunity will control this. My parents would not get it because they are afraid it will change their DNA and worried about the long term effects from the vaccine.
Confidence Vaccine Plans	Vaccine Plans Self	Kids and family member have preexisting conditions and will not do well if they receive the virus. Protect myself and others I come in contact with. It’s the only way to end the pandemic, and I need to protect my children who are too young to receive the vaccine. I believe in science but did have a questioning approach to it. The risk of COVID outweighed any risks I perceived with getting the vaccine (anaphylaxis is treatable). I got it because it was easy to schedule, free, and gave me the peace of mind to be around my older parents again. Dr. warned me that if I got infected with COVID 19 that would be a serious problem. I’m scared of COVID but I am also scared of the vaccine. I have researched each one extensively and I still believe we have somewhat rushed the process. I am young and have made it over a year and only had to quarantine once. It is not worth it to get a vaccine for me. I don’t care what someone else says, I am fully capable of making my own decisions. I travel a lot and I feel that not receiving the vaccine it would put limits on my travel. I have a previous severe adverse reaction to a vaccine, so while the risk of contracting COVID is concerning, the risk of adverse reactions is more concerning due to my personal medical history. I am vaccine damaged and extremely sensitive to the vaccine is the answer, with mutations already diminishing it effectiveness. I believe we need to focus on treatment. Choice, period. The vaccine is new and only time will give increase information on effects. I’m a child bearing aged mother that continues to want additional children and currently breastfeeding a child. I do not feel comfortable with a new vaccine and these factors. Scam and politics. Will never get the vaccine. Should take 20 plus years to test the vaccine!
Confidence	Family and Friend Vaccine Plans	Family plan to get vaccinated or have been vaccination This is the ‘safest and best way to get to “normal”. We have grandkids too young for vax, we want to protect them. Family has job commitments that necessitate lots of contact, one in health care, and they want to protect me also. They are older, work in service industry, or have preexisting conditions that make it more likely they would be hospitalized. RN wife got it only because had to for work, otherwise would not Family will not get it My aunt is a naturopath nut who’s anti-vaxer so probably won’t get it. Some of our friends don’t believe the virus is more than the flu. I wish I could put it more nicely, but it’s ignorance. Due to social media influences and political affiliation. I have a cousin who never even had chickenpox because vaccines work, but she interprets her experience as proof that she’s so healthy she doesn’t need a vaccine. Extended family members who are Trump supporters will not get the vaccine. They believe risk is over-hyped. They are conspiracy nuts. One will not because they already have it and think they can’t get it again. Also they feel they don’t have enough info on the vaccine. I have family that will not get vaccine because of what is being said on the news. It has caused a divide among the family. One family member was pregnant and did not want an unapproved vaccine while pregnant. Did not want to take something not studied in pregnant women.
Confidence Convenience	Concerns	Concerned Long term side effects. Why do healthy people need to get the vaccine Why isn’t herd immunity built on antibodies from COVID infection too? Why the push to have a vaccine when you already tested positive for antibodies in your serum. CDC and Governors change their minds daily. Know people having problems getting to a site that offers vaccines. These people have medical issues that prevent them from driving, a couple who are bedridden. They would take the vaccine. Worried it has increased my already present health conditions into something more quickly progressive (still, better than death). I don’t believe the vaccine is the best solution. I think treatment should be at the forefront of the research. It is likely that the vaccine only protects the person who gets it and causes less obvious signs of illness so vaccinated are more apt to spread it in the community.
Confidence	Type of Vaccine Matters	Based on my medical conditions and the side effects observed in others, I feel most comfortable with the Moderna. Because J&J has been proven to cause blood clotting. Does not seem worth it at all to me. Plus the vaccine still allows you to carry and transmit the virus. Don’t trust J&J. Why is it only one shot but Pfizer and Moderna are 2? Makes me think J&J isn’t as good. Wanted an mRNA vaccine, given that we had more data on those, from the vaccine push in Israel. Pfizer- because that was the one in the Israeli data. I would get Moderna, but they were offering only Pfizer. J&J is a fundamentally different kind of vaccine so if I had to pick I would take the Pfizer or Moderna, but all are fine. J&J is less effective and has more dangerous side effects. Anecdotal information is that Moderna is effective but the second shot makes more people sick. Pfizer is effective with fewer side effects. I am in the demographic risk category for TTP associated with J&J. Pfizer seems to have the best data regarding variants. Don’t want J&J—think of them for body products not medicine. J&J—I chose it because I am public facing and wanted to be fully vaccinated. The government failed public facing workers. I received Modern but was hoping to get J&J since it was a one and done. Now glad I got Modern. I am not as confident in J&J regarding the variants.
Complacency	Motivation	To be able to participate in activities that will require vaccination. Protect my patients. We are responsible outside of ourselves. Protect myself and to give my family some relief from worry. Only severe coercion would motivate me (unable to work for example). Severe tactics are unconstitutional when the vaccine has not been proven. To reduce the risk of community spread and virus mutations.

## Data Availability

Interested investigators should contact Hess for information on data sharing.
